# Latent Microsporidiosis Caused by *Encephalitozoon cuniculi* in Immunocompetent Hosts: A Murine Model Demonstrating the Ineffectiveness of the Immune System and Treatment with Albendazole

**DOI:** 10.1371/journal.pone.0060941

**Published:** 2013-04-11

**Authors:** Michaela Kotkova, Bohumil Sak, Dana Kvetonova, Martin Kvac

**Affiliations:** 1 Institute of Parasitology, Biology Centre of the Academy of Sciences of the Czech Republic, v.v.i., Ceské Budejovice, Czech Republic; 2 Faculty of Agriculture, University of South Bohemia in Ceske Budejovice, Ceske Budejovice, Czech Republic; Tulane University School of Public Health and Tropical Medicine, United States of America

## Abstract

**Background:**

Microsporidia are obligate intracellular parasites causing severe infections with lethal outcome in immunocompromised hosts. However, these pathogens are more frequently reported as latent infections in immunocompetent individuals and raises questions about the potential risk of reactivation following induced immunosuppression.

**Aims:**

To evaluate the possibility latent microsporidiosis, efficacy or albendazole, and reactivation, the authors monitored the course of *E. cuniculi* infection in immunocompetent BALB/c mice and immunodeficient SCID mice using molecular methods.

**Methods:**

Mice were per orally infected with 10^7^ spores of *E. cuniculi*. Selected groups were treated with albendazole, re-infected or chemically immunosuppressed by dexamethasone. The presence of microsporidia in the host’s organs and feces were determined using PCR methods. Changes in numbers of lymphocytes in blood and in spleen after induction of immunosuppression were confirmed using flow cytometry analysis.

**Results:**

Whereas *E. cuniculi* caused lethal microsporidiosis in SCID mice, the infection in BABL/c mice remained asymptomatic despite parasite dissemination into many organs during the acute infection phase. Albendazole treatment led to microsporidia elimination from organs in BALB/c mice. In SCID mice, however, only a temporary reduction in number of affected organs was observed and infection re-established post-treatment. Dexamethasone treatment resulted in a chronic microsporidia infection disseminating into most organs in BALB/c mice. Although the presence of *E. cuniculi* in organs of albendazole- treated mice was undetectable by PCR, it was striking that infection was reactivated by immunosuppression treatment.

**Conclusion:**

Our results demonstrated that microsporidia can successfully survive in organs of immunocompetent hosts and are able to reactivate from undetectable levels and spread within these hosts after induction of immunosuppression. These findings stress the danger of latent microsporidiosis as a life-threatening risk factor especially for individuals undergoing chemotherapy and in transplant recipients of organs originating from infected donors.

## Introduction

Microsporidia are obligate intracellular parasites that infect a wide range of vertebrate and invertebrate hosts, including humans [1]. Microscopic resistant microsporidian spores are released into the environment by infected hosts and are ubiquitous, being found in surface waters, sediments, soil, and foods [2]–[5]. The natural route of entry of the parasite into the host is by ingestion or inhalation of infectious spores, or via wounds and transplacentally [6], [7].

Although microsporidia have been known as pathogenic agents in a wide range of wild, laboratory, and domestic animals for several decades, the first case of human microsporidiosis induced by an *Encephalitozoon* spp. was recorded in 1959 [8]. Since then another 13 human-pathogenic species have been described. Among them, *Enterocytozoon bieneusi*, *Encephalitozoon cuniculi*, *E. intestinalis*, and *E. hellem*, are the most common human pathogenic microsporidia most frequently reported among immunocompromised individuals including those with acquired immune deficiency syndrome (AIDS) and transplant recipients [9].

Most of what is known about microsporidia is based on *E. cuniculi*, which commonly infects rodents in addition to humans [10]. This species was first observed in brain, spinal cord, and kidney of a rabbit with motor paralysis in 1922 [11], and subsequently described by Levaditi in 1923 [12]. *Encephalitozoon cuniculi* was also the first mammalian microsporidium that was isolated and cultured *in vitro* [13] and was reported to infect a wide range of host cells including epithelial cells, vascular endothelial cells and renal tubule cells. Spores can survive in macrophages and spread throughout the host [14] where they cause various lesions affecting the nervous system, respiratory and digestive tract, liver, peritoneum, lung, bladder, and kidney [15]–[17]. Chemotherapy of microsporidiosis is limited to only a few drugs. Albendazole inhibits microtubule assembly and is effective against several microsporidia including the *Encephalitozoon* species. Fumagilin, which is produced by *Aspergillus fumigatus*, is more broadly effective against *Encephalitozoon* spp. and *E. bieneusi* [18]. Similarly, protease inhibitor (antiretroviral) therapy indirectly leads to resolution of microsporidiosis in HIV patients through restoration of immune competence [19].

Immunobiology of microsporidial infections is primarily studied in immunocompetent BALB/c mice and immunodeficient SCID mice [20]–[22]. In immunocompetent BALB/c mice, the *E. cuniculi* infection remains asymptomatic as long as parasite multiplication and the host immune response are balanced [20]. On the contrary, in athymic or SCID mice, microsporidia infect various internal organs with probable lethal outcome [21], [22]. In immunocompetent humans, a short acute diarrheal phase is probably followed by asymptomatic infection. However, chronic malabsorbtive diarrhea and systemic disease can develop in immunocompromised individuals [23].

Chronic microsporidia infections caused by *E. cuniculi* in immunocompetent individuals are generally asymptomatic, probably reflecting a balanced parasite-host relationship. It appears that elimination of microsporidia requires chemotherapeutic intervention. The efficacy of albendazole in eliminating microsporidia from immunocompetent hosts has not been addressed using *in vivo* experimental infections. All previous studies were focused only on extending the survival time of hosts [24]–[26]. This approach ignored the possible survival of microsporidia in albendazole-treated individuals and the development of latent infection. Latent microsporidiosis in immunocompetent hosts could lead to infection relapse following immunosuppression. Thus, the present study was designed to determine the effectiveness of treatment against the infection caused by *E. cuniculi* and the potential re-activation and re-dissemination of infection after artificial immunosuppression. Our findings bring a new perspective to neglected, latent microsporidiosis and enhance our understanding of the epidemiology and natural history of microsporidiosis.

## Materials and Methods

### Ethics Statement

All of the experimental procedures were conducted in accordance with the law of the Czech Republic on the use of experimental animals, safety and use of pathogenic agents. The study was approved by the Institute of Parasitology, Biology Centre of the Academy of Sciences of the Czech Republic and Institutional and National Committees (protocols no. 070/2010).

### Experimental Animals

Adult SCID mice (strain C.B-17) of the BALB/c background and BALB/c mice were originally obtained from Charles River, Sulzfeld, Germany and bred in plastic cages with sterilized wood-chip bedding situated in IVC Air Handling Solutions (Techniplast, Italy) with high-efficiency particulate air (HEPA) filters. The experimental 8-week-old animals were housed in plastic cages with sterilized wood-chip bedding situated in flexible film isolators (BEM Znojmo, Czech Republic) with HEPA filters. All mice were supplied with a sterilized diet (TOP-VELAZ Praha, Czech Republic) and sterilized water *ad libitum*.

### Parasite

The spores of *E. cuniculi* strain EC2 were originally isolated from a dexamethasone-treated laboratory mouse [26] and were grown *in vitro* in Green monkey kidney cells (VERO, line E6) maintained in RPMI-1640 medium (SIGMA) supplemented with 2.5% heat-inactivated fetal bovine serum. Spores were isolated and purified from cells by centrifugation over 50% Percoll (SIGMA) at 1,100×*g* for 30 min and washed three times in sterilized deionised water before storing in sterilized deionised water supplemented with antibiotics (SIGMA, 100 U/ml penicillin, 100 µg/ml streptomycin, and 2.5 µg/ml amphotericin B) at 4°C. The spores were washed in sterilized deionised water before use.

### Drugs Application

Aldifal (MEVAK NITRA, SR) containing 100 g of albendazole in 1000 ml, was dosed for treatment of microsporidiosis as follows: a total 0.2 mg of albendazole dissolved in 200 µl deionised sterilized water was applied daily per orally (p.o.) by intragastric gavage per animal. Dexamethasone (0.85 mg/ml) (Intervet) was used for immunosuppression. Dexamethasone (35 µg dissolved in 160 µl PBS) was applied daily i.p (intraperitonealy) per animal.

### Experimental Protocols

#### Experiment no. 1

Course of infection caused by *E. cuniculi* in BALB/c and SCID mice. Groups of 69 BALB/c and 69 SCID mice were infected p.o. with 10^7^ *E. cuniculi* spores in 0.2 ml of sterilized deionised water by intragastric gavage. Thirty BALB/c mice and 30 SCID mice were treated daily p.o. with albendazole from 28 to 42 days post infection. Moreover, 42 mice of both non-infected SCID and BALB/c were used as negative controls.

#### Experiment no. 2

**Figure 1:**
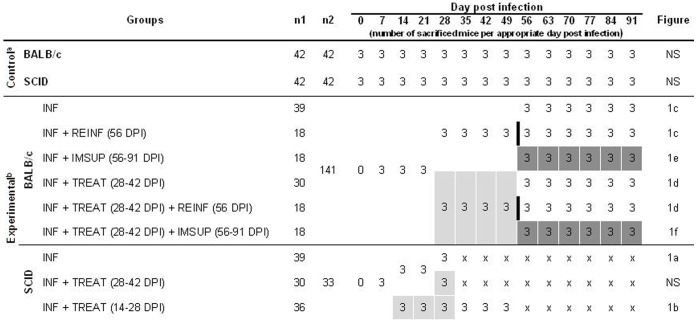
Design of experiments. **^a^**inoculation with 200 ul sterilized deionised water; **^b^**infection with 10^7^ *E. cuniculi* spores in 0.2 ml of sterilized deionised water; INF – infection; REINF – reinfection (black column); IMSUP – dexamethasone immunosuppression (highlighted in dark grey); TREAT – albendazole treatment (highlighted in light grey); **n1–** number of used animals; **n2**– number of dissected animals; **NS** – not shown; **x** – not observed due to mouse death; **DPI** – day post infection;

Simulation of re-infection and determination of the albendazole efficacy after dexamethasone-induced immunosuppression;

Groups of 141 BALB/c and 75 SCID mice were infected p.o. with 10^7^ *E. cuniculi* spores in 0.2 ml of deionised water by intragastric gavage. Thirty-six SCID mice were treated daily p.o. with albendazole from 14 to 28 days post infection (DPI). Thirty BALB/c mice were treated with daily with albendazole 28–42 DPI. Eighteen albendazole-treated BALB/c mice and 18 BALB/c mice in the chronic stage of infection (without albendazole treatment) were re-infected p.o. with the same dose of spores of *E. cuniculi* 56 days after the first infection. Eighteen albendazole-treated BALB/c mice and 18 BALB/c mice in chronic stage of infection were immunosuppressed by daily dexamethasone treatment from 56 to 91 DPI. Thirty-nine BALB/c mice in the chronic stage of infection were used as positive controls for monitoring the course of infection. Forty-two non-infected SCID and 42 BALB/c mice served as negative controls in each type of experiment. The study design of all experiments is presented in the Fig. 1.

### Assessment of Infections

Fecal samples were obtained daily from each mouse and stored at −20°C prior to DNA isolation. Mortality and morbidity were recorded daily. Three mice from each group were euthanized every seventh day post infection (see Fig. 1). Sterile samples were obtained as follows: urine by bladder catheterization, blood from retroorbital sinus, peritoneal lavage with cold sterile PBS, and organ samples (stomach, duodenum, ileum, jejunum, caecum, colon, liver, spleen, kidney, bladder, lung, heart, and brain). Each organ was removed using a different pair of sterile dissection tools and stored at −20°C prior to DNA isolation. In addition, half of the spleen and 75 µl of the blood from immunosuppressed BALB/c mice, BALB/c mice without infection, and BALB/c mice after albendazole treatment were used for lymphocytes enumeration by flow cytometry. All samples were used for molecular analysis (see below).

### DNA Isolation

Fecal and organ samples were homogenized by bead disruption using a FastPrep®–24 Instrument (MP Biomedicals, CA, USA) and 0.5 mm glass beads (Biospec Products, Inc., Bartlesville, OK, USA) at the speed of 5.5 m/s for 1 min. Total DNA was extracted using commercial column-based isolation kits, QIAamp® DNA Stool Mini Kit and DNeasy Blood & Tissue Kit, respectively (both QIAGEN, Hilden, Germany). Acquired DNA was stored at −20°C.

### PCR Amplification

We used a nested PCR protocol to amplify a partial sequence of SSU rRNA using microsporidia-specific primers previously described by De Bosscuere et al. [27] and Katzwinkel-Wladarsch et al. [28]. The upstream primers M2F (CGG AGA GGA AGC CTT AGA GA) and MFNest (GAG AGA TGG CTA CTA CGT CCA AGG) were targeted to the 3′ region of the SSU coding segment of *E. cuniculi*. The downstream primers M2R (ATA GTG ACG GGC GGT GTG T) and MSP1R (ACA GGG ACM CAT TCA) were targeted to the 5′ region of the coding segment of *E. cuniculi*. For the primary PCR step, the PCR mixture contained 1× PCR buffer, 3 mM MgCl_2_, 0.2 mM each dNTP’s, 1 U *Taq*, 1 µl BSA (10 mg/ml), and 200 nM each primer. For the secondary PCR step, the PCR mixture was identical except that BSA was excluded. DNA obtained from spores of *E. cuniculi* grown *in vitro* in VERO E6 was used as a positive control. Water was used instead of template as the negative control. For both PCR steps a total of 35 cycles, each consisting of 94°C for 45 s, 58°C for 45 s, and 72°C for 60 s, were performed. Initial incubation at 94°C for 3 min., final extension at 72°C for 7 min., and final soak at 4°C were included. PCR products were visualized on a 1% agarose gel containing 0.2 µg/ml ethidium bromide. One to three randomly selected positive samples from each animal were sequenced and compared to the sequence of the isolate in the inoculum. If one of the samples originating from triplicate mice was found positive, the organ was considered positive.

### Flow Cytometry Analysis

Halves of each spleen and 75****µl of blood from immunosuppressed BALB/c mice after albendazole treatment and immunosuppressed BALB/c mice in chronic stage of infection were used for flow cytometry analysis. Halves of each spleen and 75****µl of blood of BALB/c mice after albendazole treatment, and BALB/c mice in chronic stages of infection were also used for flow cytometry analysis as controls.

Whole splenocyte suspensions were prepared by gentle extrusion through plastic sieves into cold RPMI 1640 medium. Cell suspensions were washed three times in RPMI 1640 medium by centrifugation at 160×*g* for 10 min at 4°C. The viability of spleen cells was assessed by Trypan blue exclusion immediately after their recovery [29].

A total of 75 µl of blood was collected into 1 ml PBS containing 5 µM EDTA and mixed immediately to prevent clotting. Red blood cells were lysed using a buffered 0.84% ammonium chloride solution and incubated in a water bath at 37°C for 3 min. Cells were washed three times by centrifugation at 160×*g* for 10 min at 4°C with FACS buffer (PBS supplemented with 0.2% gelatine and 0.01% sodium azide). The pellet from the final wash was resuspended in 100 µl FACS buffer.

The levels of leukocytes in cell preparations from blood and spleens were analysed by flow cytometry. Samples (0.5×10^6^ spleen cells and all obtained blood cells) were incubated for 30 min at 4°C with specific monoclonal antibodies (all obtained from PharMingen, San Diego, CA, USA) against surface antigens diluted in FACS buffer. The following monoclonal anti-mouse antibodies (MAbs) were used: anti-CD45 MAb, anti-CD3 MAb, anti-CD4 MAb, anti-CD8 MAb and anti-CD19 MAb. Labeled cell samples were analyzed on a BD FACSCanto II flow cytometer (BD Biosciences, USA) equipped with two lasers with excitation wavelengths of 488 and 633 nm. The labeled cell populations were analyzed using DIVA software.

### Statistical Analysis

The statistically significant differences in survival time of albendazole-treated and non-treated SCID mice, and the differences between the decline in lymphocytes in groups of immunosuppressed mice were analyzed by non-parametric Mann-Whitney *U* test. Both tests were performed using Statistica 6.0 software (StatSoft CR, Praha, Czech Republic).

## Results

**Figure 2:**
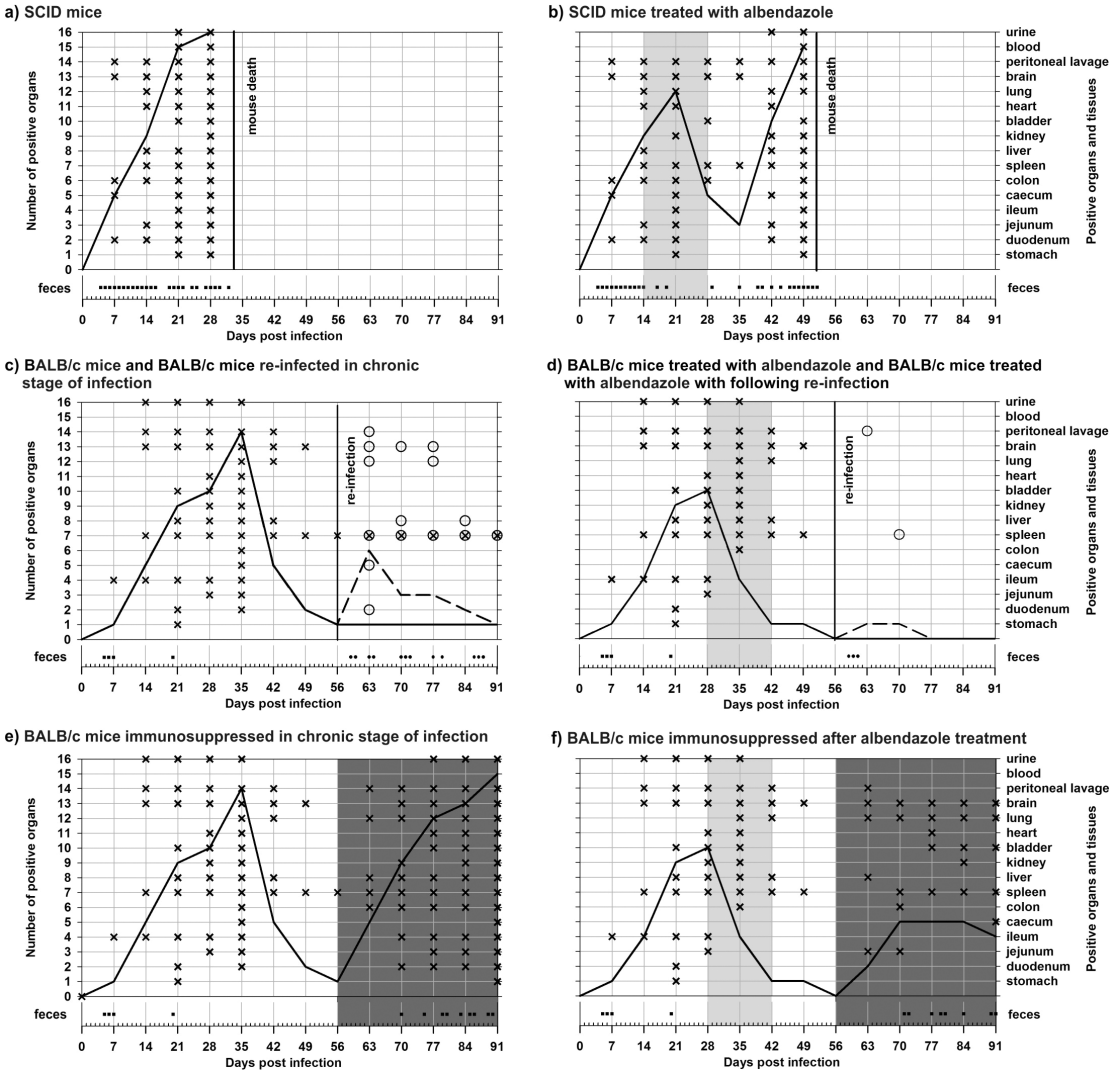
Course of *Encepahlitozoon cuniculi* genotype II infection, including pattern of spore shedding and dissemination of infection to selected organs and tissues. **a)** SCID mice, **b)** SCID mice treated with albendazole, **c)** BALB/c mice and BALB/c mice re-infected in chronic stage of infection, **d)** BALB/c mice treated with albendazole and BALB/c mice treated with albendazole with following re-infection, **e)** BALB/c mice immunosuppressed in chronic stage of infection, **f)** BALB/c mice immunosuppressed after albendazole treatment. **Light-gray field** – albendazole treatment; **dark-gray field** – dexamethasone immunosuppression; **black line** – course of *E. cuniculi* infection; **black dash line** - course of *E. cuniculi* re-infection; **cross** – *E. cuniculi* positive organ during primarily infection; **ring** – *E. cuniculi* positive organ during re-infection; **black square** – spores shedding during primarily infection; **black circle** – spores shedding during re-infection.

The experimental p.o. inoculation of *E. cuniculi* caused a severe, fatal disease in SCID mice, which was characterized by the dissemination of microsporidia into all organs and tissues within 4 weeks. Coprological examination of mice revealed microsporidia almost every day from day 4 post-infection (Fig. 2a). The mean survival time (MST) of infected SCID mice was 33±0.5 days.

The treatment of infected SCID mice with albendazole from 14 to 28 DPI extended survival and resulted in the disappearance of microsporidia from numerous organs immediately after treatment introduction (Fig. 2b). While microsporidia were detected in fecal samples every day from 4 DPI, no spores were found in feces during the second week of treatment. After discontinuation of albendazole treatment, however, the parasite re-disseminated and mice died within 3 weeks (MST = 51.5±1.3 days). In addition, one week after interruption of albendazole treatment spores were present again almost every day in feces.

In contrast, treatment of infected SCID mice in acute phase with albendazole from day 28 post-infection had no effect and did not prevent mortality. The mean survival time was 32.1±1.0 days (data not shown).

Microsporidiosis caused by *E. cuniculi* in BALB/c mice has a progressive course characterized by the dissemination of microsporidia into almost all organs within 35 days p.i. After this acute stage, microsporidia disappeared from most organs with the exception of the spleen, which was positive up to 91 DPI when the experiment was terminated (Fig. 2c). Fecal samples were positive 5 to 7 DPI and 20 DPI.

Albendazole treatment of BALB/c mice, which was carried out 28 to 42 DPI, had a noticeable effect after one week of treatment. The disappearance of the parasite from the organs is shown in Fig. 2d. All organs and tissues were negative for microsporidia two weeks after the termination of albendazole therapy. Fecal samples examined for the presence of microsporidia were positive only 5 to 7 DPI.

Re-infection of BALB/c mice in chronic stage of infection led to reappearance of the parasite in many organs including the brain, lungs, spleen, and liver. Of these, only the spleen remained positive till 91 DPI as shown in Fig. 2c. Microsporidial DNA was detected in fecal samples after re-infection at irregular intervals. In contrast to re-infected BALB/c mice, re-infection of albendazole-treated BALB/c mice did not lead to extensive dissemination of microsporidia (Fig. 2d). *E. cuniculi* was detected only in peritoneal lavage one week after re-infection (63 DPI), and in the spleen 14 days after re-infection (70 DPI). No other organs or tissue were found positive for the parasite following re-infection. *E. cuniculi* spores were presented in feces 3 to 5 days after re-infection.

After one week of daily dexamethasone applications to two groups of BALB/c mice, the levels of lymphocytes had decreased by as much as 96% in the blood and by 78% in spleen compared to negative controls (data not shown). The mean (±SD) decrease of CD8+ T-cells detected in the spleen was 69.4% (±1.25%), CD4+ T-cells 62.6% (±1.08%), CD3+ T-cells 66.1% (±1.42%), CD19+ T-cells 77.24% (±3.08%) and 75.81% (±1.55%) in case of CD45+ T-cells (*P*<0.05). The mean (±SD) decrease of CD8+ T-cells detected in the blood was 91.88% (±1.44%), CD4+ T-cells 88.75% (±0.5%), CD3+ T-cells 90.02% (±0.83%), CD19+ T-cells 96.1% (±0.23%), and 81.36% (±1.46%) in the case of CD45+ T-cells (*P*<0.05). The counts of lymphocytes after immunosuppression in BALB/c mice after albendazole treatment and BALB/c mice in chronic stage of infection were almost equal. Lymphocyte levels remained low during the remainder of the immunosuppression period.

Application of dexamethasone in BALB/c mice in the chronic stage of infection caused expansion of the parasite into many organs after one week that continued for several weeks (Fig. 2e). Microsporidia were found in all organs by week 5 post immunosuppression (91 DPI). Moreover, microsporidia were again alternately detected in feces from day 14 post immunosuppression.

Application of dexamethasone in BALB/c mice following treatment with albendazole resulted in parasite dissemination to many organs, as well (Fig. 2f). In particular, the brain and lungs were positive for microsporidia at each time point tested after immunosuppression. However, the extent of parasite dissemination in albendazole-treated, immunosuppressed BALB/c mice was lower than that observed in immunosupressed BALB/c mice without albendazole treatment.

## Discussion

*Encephalitozoon cuniculi* is the most-studied species of microsporidia and the majority of information about the pathogenesis of microsporidiosis is derived from studies of this organism. Because *E. cuniculi* has a low host specificity and its spores are resistant to adverse environmental conditions, man can easily get infected with this parasite, for example, through water and food contaminated by feces or urine of infected animals (zoonotic transmission) [6], [7], [30].

Microsporidia in humans are considered opportunistic pathogens, because they primarily cause disease when the host’s immunity is reduced and so the parasite can easily overspread. The first case of human microsporidiosis was recorded in 1959 [8]. The number of cases increased during the 1990s coincident with the rising HIV/AIDS pandemic. Most microsporidial infections caused by *E. cuniculi* are in immunocompromised patients (HIV positive individuals, patients undergoing organ transplantation or patients with idiopathic CD4+ T lymphocytopenia) [31]. When the T-cell count falls below 100/µl blood, infection manifests itself as acute microsporidiosis, often with systemic involvement [17], [32]. Over the decades, several reviews on non-specific and adaptive immune responses involved in the fight against microsporidial infection have been published [33]–[35]. It is generally accepted that a protective immune response against this parasite is mediated by cytotoxic CD8+ T-lymphocytes [36] and their activation does not appear to be dependent upon CD4+ T-cells [37], [38]. It was found that IFN-γ is the primary mechanism that mediates partial protection of SCID mice in the absence of CD4+ and CD8+ T-lymphocytes [34]. This cytokine can enhance the cytotoxic activity of natural killer cells and activate macrophages to effectively kill phagocyted microsporidial spores [39]. Moreover, activated macrophages also produce IFN-γ, which amplifies macrophage activation. Furthermore, T-cell-dependent B-cell activation for antibody production is also important in protection against microsporidia [40], [41]. Recent results of Sak et al. [42] showed that *E. cuniculi* represents the vast majority of the microsporidial species found in the healthy population in the Czech Republic. Moreover, the majority of examined individuals were without any clinical symptoms. Our results imply that a competent immune response is unable to fully eliminate the infection even if there are no clinical signs. The question remains how microsporidia are able to survive in sufficient quantities in the host for a long time despite an activated immune system.

The course of infection caused by *E. cuniculi* in an immunocompetent host can be easily demonstrated in murine models such as in BALB/c mice. Furthermore, the SCID mouse is a suitable model for studying the pathogenesis and potential chemotherapeutics with anti-microsporidial effects [21]. As the present and previous reports show, microsporidiosis in hosts without a functional immune system has a rapid course with fatal consequence [36], [40], [41]. In contrast with general assumptions (see above), our results conclusively demonstrate that *E. cuniculi* remain in some organs of immunocompetent hosts (BALB/c mouse) for a long time. In such cases, these organs can serve as constant sources of microsporidial spores.

The presented data highlight the potential dangers of latent microsporidiosis. During the last decade, microsporidia have been more frequently reported in immunocompetent individuals, producing asymptomatic infections [42], [43]. As proven by our experiments, these latent infections may be reactivated during immunosuppression and the carrier can be a source of infection for at-risk groups. A recent case report described microsporidial keratoconjunctivitis that was transmitted by the donor corneal graft [44]. Latent microsporidiosis also can be dangerous for the carrier himself if undergoing chemotherapy for cancer or other immunosuppressive therapies. In addition, microsporidia can also cause serious disease in immunocompetent hosts [45].

Albendazole or its other derivates are commonly used for treatment of microsporidiosis caused by *Encephalitozoon* spp. [18]. The efficacy of this therapy was studied here using sensitive molecular detection of parasites for the first time. Treatment extended the survival of SCID mice and appeared to eliminate *E. cuniculi* from BALB/c mice. However, microsporidiosis was surprisingly re-activated in albendazole treated BALB/c mice after dexamethasone-induced immunosuppression. This implies that the population of *E. cuniculi* organisms that was not detectable by PCR remained intact after albendazole treatment and this population expanded and disseminated following subsequent immunosupression. Although the number of affected organs was not as high as that in infected SCID mice, the evidence that an undetectable infection can be reactivated is of major significance for public health. It is perhaps unsurprising that *E. cuniculi* spread rapidly following immunosuppression and the number of affected organs was similar to that observed in SCID mice. Lallo et al. [46] reached similar conclusions, when cyclophosphamide-immunosuppressed mice with *E. cuniculi* infection showed clinical symptoms of acute, lethal and widespread microporidiosis affecting the liver, lungs, intestines, kidneys and brain. In contrast cyclosporin-immunosuppressed mice inoculated with *E. cuniculi* developed mild, chronic infection with a few clinical manifestations and histological lesions.

Recent data would suggest that the incidence of microsporidial infections is much higher than previously reported [47], [48]. The true incidence is difficult to estimate due to the presence of asymptomatic carriers [42], [43], [49], [50]. The results observed by Sak et al. [42], [43] demonstrated the high prevalence of microsporidia in naturally infected immunocompetent humans and various species of animals suggesting that immune mechanisms of defense against microsporidia do not completely clear the organisms. Moreover, our data further support this interpretation since immunecompetent BALB/c mice infected only once or mice re-infected after albendazole treatment shed spores in feces for only a few days despite persistent microsporidia within the host that reactivated after dexamethasone treatment. In contrast, re-infection of BALB/c mice during chronic infection led to intermittent shedding of spores in feces for a long period. It follows that a single test can give false negative results in up to 70% of cases [42], [43].

Consequently, results of our experiments can be implemented into practice; individuals with latent microsporidiosis may be at risk if they become immunocompromised, and should be tested for microsporidia if undergoing chemotherapy, transplantation, or other therapy requiring a suppression of the immune system. Moreover, it is necessary to repeatedly examine stool specimens for a few days in order to detect intermittent shedding of microsporidial spores. Unfortunately, microsporidia are often overlooked and underdiagnosed because of the lack of an adequately sensitive and specific method.

In conclusion, our study shows the induction of acute and subsequent chronic infection with microsporidia in immunocompetent laboratory mice, and successful re-activation and re-dissemination of the infection after artificial immunosuppression. Understanding how microsporidia survive in their hosts despite a competent immune system can help explain the emergence of latent microsporidiosis. This would also answer questions concerning the possibility of repeated re-infections, relapse after immunosuppression, efficacy of immune system and use of anti-microsporidial treatment and may lead to introduction of reliable methods for testing the presence of microsporidia infections.
